# Elevated Serum Interleukin-34 Level in Patients with Systemic Lupus Erythematosus Is Associated with Disease Activity

**DOI:** 10.1038/s41598-018-21859-z

**Published:** 2018-02-22

**Authors:** Huan Huan Xie, Hui Shen, Li Zhang, Mei Ying Cui, Li Ping Xia, Jing Lu

**Affiliations:** 1 Department of Rheumatology, 1st Affiliated Hospital of China Medical University, Shen Yang, China; 2 Department of Rheumatology, Dazhou Central Hospital, Da Zhou, China

## Abstract

We measured the interleukin-34 (IL-34) level in sera from patients with systemic lupus erythematosus (SLE) and discoid lupus erythematosus (DLE) using an enzyme-linked immunosorbent assay (ELISA). Blood tests, including assays to determine C-reactive protein (CRP), complement (C) 3, C4, immunoglobulin (Ig) A, IgG, IgM, anti-double-stranded DNA antibody (Anti-dsDNA Ab) and hemoglobin (Hb) levels and white blood cell (WBC) and platelet (PLT) counts, were performed using standard methods. Lupus nephritis (LN) was diagnosed according to the American College of Rheumatology (ACR) renal criteria. The SLE disease activity was scored using the SLE Disease Activity Index (SLEDAI). Among the 110 SLE cases, IL-34 could be detected in 79 cases (71.8%). IL-34 was barely detected in the control group. The serum level of IL-34 was significantly higher in the SLE group. No change was observed in the serum IL-34 concentration in the SLE patients regardless of LN status. Correlations were observed between the serum IL-34 level and the disease activity parameters. The SLE patients with detectable IL-34 levels had higher SLEDAI and IgG concentrations and lower C3 and Hb levels than patients with undetectable IL-34 levels. Therefore, IL-34 could be a potential disease activity marker for SLE.

## Introduction

Systemic lupus erythematosus (SLE) is a chronic systemic autoimmune disease characterized by a dysregulated autoantibody production and complement activation, resulting in multiple system and organ damage, particularly in the kidneys, blood system and central nervous system^1^. The treatment of SLE patients with glucocorticoids and immunosuppressive drugs has a significant impact on the outcome of this disease.

Recently, many cytokines have been shown to play important roles in the pathogenesis of SLE, and several biological products have been used in SLE patients. In 2011, the US Food and Drug Administration (FDA) approved belimumab, which is a fully human monoclonal antibody against B lymphocyte stimulator (BLyS), as a new biological drug for the treatment of SLE^2^. Several other potential biological targets are currently under investigation^3^. However, using the current therapeutic strategies, only a very small proportion of SLE patients achieve long-term remission^4^. Thus, more effective and safer drugs are needed to reduce the disease activity and achieve long-term remission.

Interleukin (IL)−34 is a newly discovered cytokine that has no significant amino acid sequence homology to other cytokines^5^. Currently, knowledge regarding this cytokine is limited. IL-34 shares a common receptor with macrophage-colony stimulating factor (M-CSF)^6^. Because IL-34 is an alternative ligand of the colony-stimulating factor-1 receptor (CSF-1R), IL-34 binds to CSF-1R and promotes the differentiation and proliferation of lymphocytes and the expression of cytokines, leading to inflammatory lesions and autoimmunity^5,7^. The serum level of IL-34 is elevated in rheumatoid arthritis (RA) patients^8,9^.

IL-34 can induce the expression of IL-6, interferon γ-inducible protein (IP) 10 and monocyte chemoattractant protein-1 (MCP-1) in human whole blood^7^. IL-6^10,11^, IP10^12^ and MCP-1^13^ participate in the pathogenesis of SLE.

The expression of IL-34 was increased in a study investigating kidney tissues from a murine model of lupus nephritis (LN)^14^. However, whether IL-34 is released into the circulation in SLE patients and the relationship between IL-34 and the clinical parameters remain unclear. We hypothesized that IL-34 might play a role in SLE.

## Results

### Clinical features of patients with SLE

In total, 110 SLE patients were recruited. Their clinical characteristics are summarized in Table 1. LN was found in 63 of the 110 patients.

**Table 1:** Clinical data of the SLE patients.

Characteristics	Values		
	SLE	DLE	NC
Age (yrs)	39.5 ± 10.1	38.7 ± 12.9	36.8 ± 9.7
Sex (F/M)	107/3	30/1	53/2
Disease duration (yrs)	5.73 ± 5.37	1.45 ± 1.19	ND
ESR (mm/h)	44.2 ± 23.0	24.6 ± 18.7	ND
CRP (mg/L)	21.67 ± 48.27	10.61 ± 11.70	ND
LN (with/without)	63/47	0/31	ND
C3 (g/L)	0.62 ± 0.31	0.70 ± 0.29	ND
C4 (g/L)	0.13 ± 0.09	0.24 ± 0.19	ND
IgG (g/L)	16.97 ± 0.72	12.10 ± 0.68	ND
IgA (g/L)	3.13 ± 0.16 2	78 ± 0.21	ND
IgM (g/L)	1.09 ± 0.10	1.07 ± 0.18	ND
Hb (g/L)	108.30 ± 2.28	12.78 ± 2.56	ND
PLT (10^9^/L)	187.90 ± 11.08	200.04 ± 31.28	ND
WBC (10^9^/L)	5.57 ± 0.32	6.23 ± 1.62	ND
Anti-dsDNA (IU/mL)	50.56 (6.91–778.45)	30.17 (5.89–168.23)	ND
SLEDAI	8 (0–24)	ND	ND

### Patients with SLE had elevated IL-34 levels

**Figure 1:**
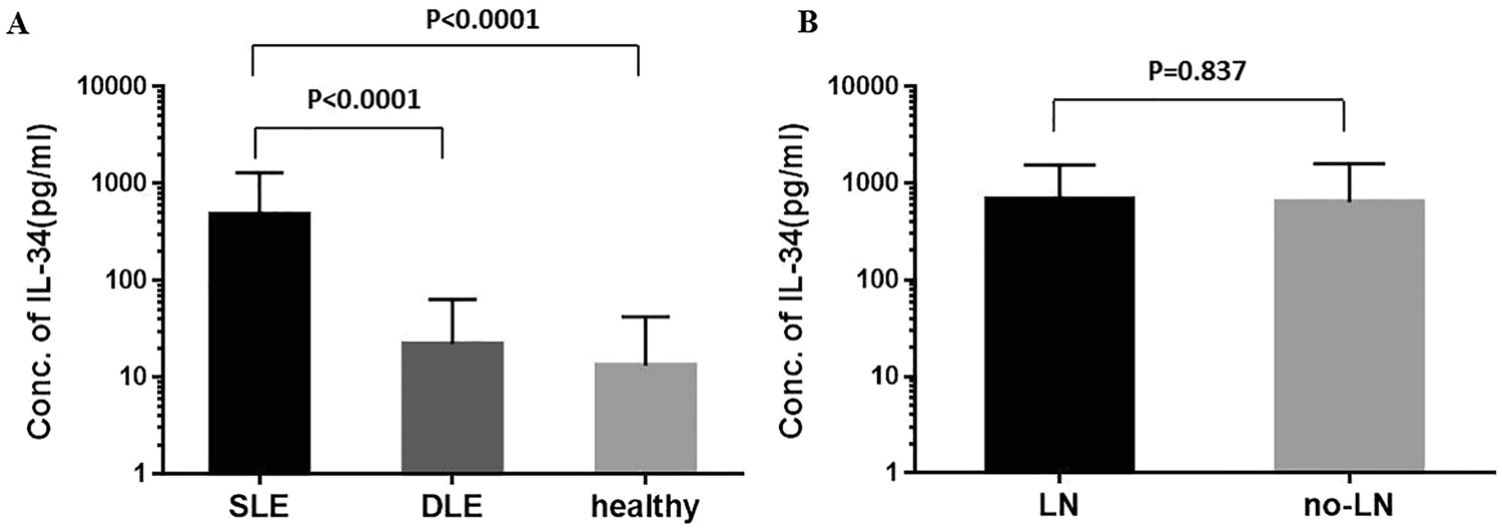
Elevated IL-34 levels in the patients with SLE.

While IL-34 was nearly undetectable in the serum from the DLE patients (9.6%) and healthy controls (5.5%), IL-34 was detected in 79 of the 110 SLE patients (71.8%). The serum IL-34 level was significantly higher in the patients with SLE than that in the healthy controls (p < 0.001) (Fig. 1A).

The association between IL-34 and LN was explored. LN was found in 63 of the 110 patients. No difference was observed in the IL-34 level regardless of the renal disease status (Fig. 1B).

### Serum IL-34 level and disease activity in SLE patients

**Figure 2:**
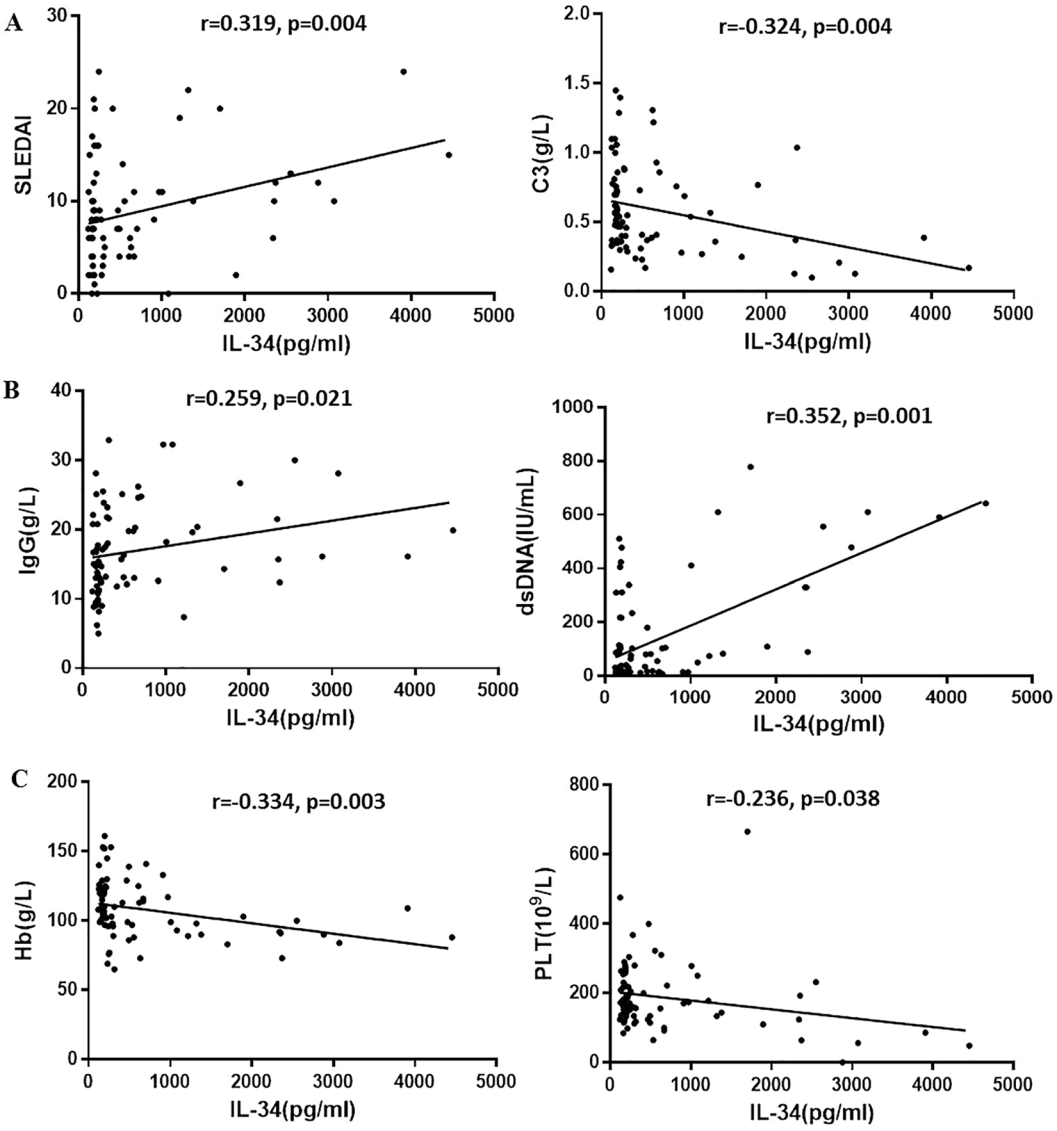
Serum IL-34 level and disease activity in the SLE patients.

A significant positive correlation was observed between the IL-34 levels and the disease activity marker SLEDAI (r = 0.319, p = 0.004) (Fig. 2A) but not with CRP. In contrast, a statistically significant negative correlation was observed between IL-34 and C3 in the SLE patients (r = 0.324, p = 0.004) (Fig. 2A). Thus, the IL-34 level could be correlated with the SLE disease activity. Furthermore, the serum IL-34 level was correlated with IgG and anti-dsDNA antibody production in SLE patients (r = 0.259, p = 0.021; r = 0.352. p = 0.001, respectively) (Fig. 2B). Thus, IL-34 might be associated with antibody production in SLE pathogenesis.

Because hematological changes are quite common in SLE patients, the IL-34 level is significantly negatively associated with Hb and PLT (r = 0.334, p = 0.003; r = 0.236, p = 0.038, respectively) (Fig. 2C). No correlation was observed between the IL-34 level and the WBC count.

According to a multivariate linear regression analysis, SLEDAI and hemoglobin were independently correlated with the circulating levels of IL-34 in the SLE patients (ß = 0.319, p = 0.008 and ß = −0.275, p = 0.023) (Table 2).

**Table 2:** Multivariate linear regression analysis of IL-34 in patients with SLE.

	ß	p
SLEDAI	0.319	0.008
C3	−0.096	0.098
IgG	0.086	0.103
dsDNA	0.099	0.061
Hb	−0.275	0.023
PLT	−0.073	0.105

### Differences in SLE clinical indices between the IL-34-positive and -negative groups

Of the 110 SLE patents, IL-34 could be detected in 79 patients. These patients were allocated into 2 groups, i.e., IL-34-positive group (IL-34^+^ group, IL-34 detectable SLE patients) and the IL-34-negative group (IL-34^−^ group, IL-34 undetectable SLE patients), based on their IL-34 status.

**Figure 3:**
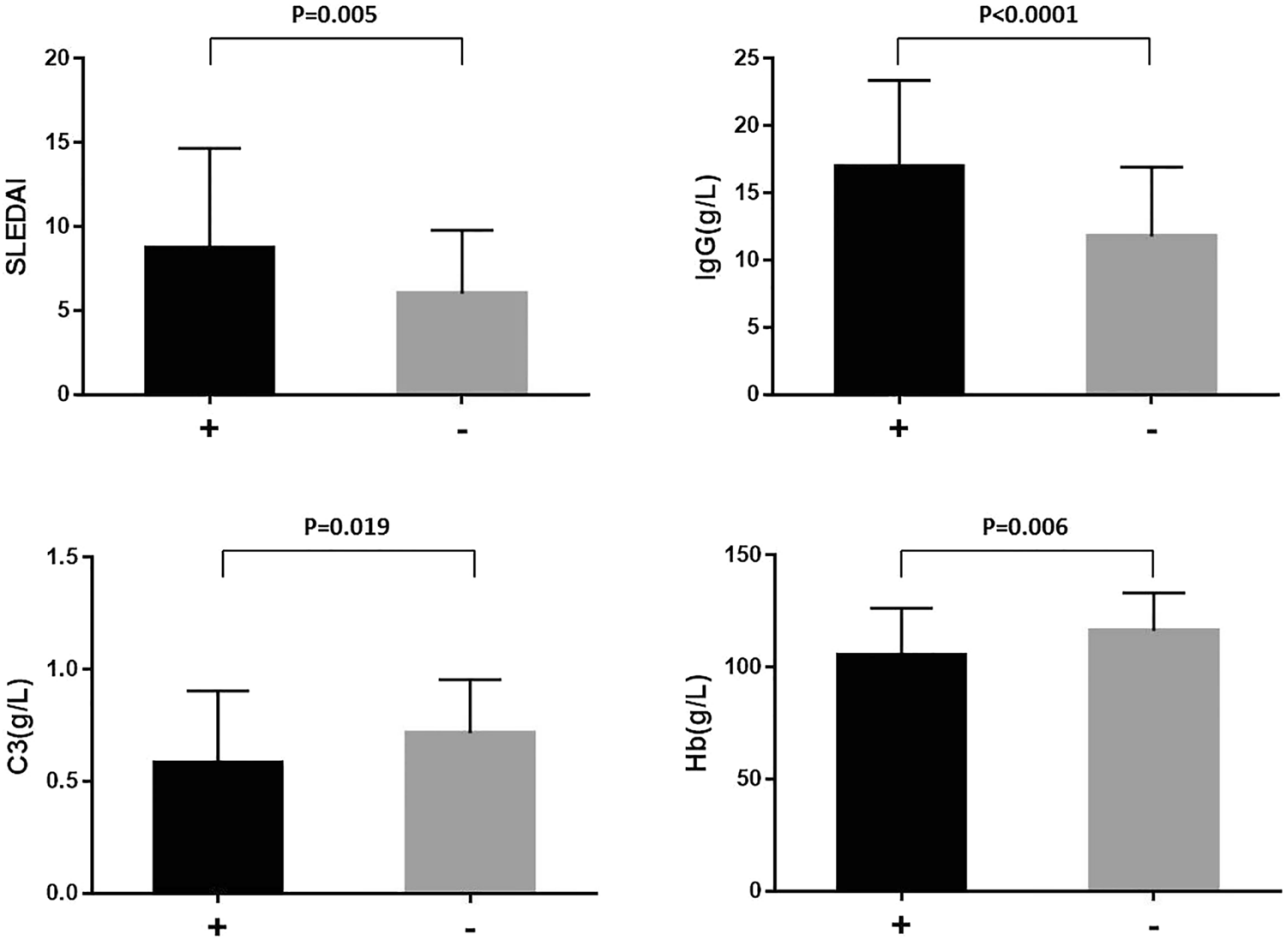
Differences in SLE clinical indices between the IL-34-positive and -negative groups.

The levels of SLEDAI and IgG in the IL-34^+^group were much higher than those in the IL-34^−^group (SLEDAI: 8.75 ± 0.66 vs 6.00 ± 0.68, p = 0.005; IgG: 16.97 ± 0.72 vs 11.79 ± 0.92 g/L, p < 0.0001) (Fig. 3A), while the levels of C3 and Hb in the IL-34^+^ group were much lower than those in the IL-34^−^ group (C3: 0.58 ± 0.04 vs 0.72 ± 0.04 g/L, p = 0.019; Hb: 105.3 ± 2.37 vs 116.3 ± 3.04 g/L, p = 0.006) (Fig. 3B).

No difference was found in PLT between the two groups.

## Discussion

This study is the first to show that the IL-34 level is elevated in SLE patients and correlated with the disease activity. In this study, we reported that the serum IL-34 level was significantly elevated in the SLE patients compared to that in the healthy controls. Among the SLE patients with or without LN involvement, no statistically significant difference was observed in the IL-34 level. However, according to Bethunaickan R, the expression of IL-34 is increased in mice with LN^14^, and IL-34 might play a role in the pathogenesis of LN. The current data are not sufficient to reach such a conclusion, and more evidence is required.

Moreover, in our study, the IL-34 level is significantly positively correlated with the disease activity as assessed by the SLEDAI. The serum C3 level decreases in SLE patients, particularly in patients with an active disease status. In this study, the serum IL-34 level was correlated with the decreased C3 level. Furthermore, the levels of SLEDAI in the IL-34^+^group were much higher than those in the IL-34^-^group. In contrast, the C3 level in the IL-34^+^group was much lower. Altogether, the serum IL-34 level might be associated with the disease activity in SLE.

Furthermore, we showed correlations among IL-34, IgG and anti-dsDNA Ab. Thus, IL-34 might be associated with antibody production in SLE pathogenesis.

Because SLE is a highly heterogeneous disease, characterizing subgroups of SLE patients with specific disease phenotypes could help clarify the pathogenesis of SLE. Cytokine measurements might lead to the characterization of these pathways and the identification of potential therapeutic targets. Hematological involvement is quite common in patients with SLE. In this study, the IL-34 level was negatively correlated with hemoglobin and exhibited a weak negative association with platelets. However, in the subsequent analysis of the groups with the IL-34+ & IL-34− SLE patients, a difference in hemoglobin, but not in platelets, was observed. Thus, IL-34 may be associated with anemia in SLE patients. However, more studies are needed to clarify whether IL-34 plays a role in the hematological changes observed in SLE patients.

Elevated concentrations of inflammatory mediators are characteristic of autoimmune diseases with chronic or recurrent inflammation. IL-34 plays pivotal roles in the proliferation and differentiation of mononuclear phagocyte lineage cells and osteoclastogenesis and is necessary for the maintenance of Langerhans cells after the resolution of inflammation^14,15^. Recently, IL-34 has been shown to play important roles in the pathogenesis of RA^8,9^. IL-34 is overexpressed in the inflamed salivary glands of patients with Sjogren’s syndrome (SS)^16^. Furthermore, IL-34 may play a role in cancer. IL-34 promotes tumor progression and metastatic processes in osteosarcoma via the induction of angiogenesis^17^. However, our study has limitation for not including a disease control group, such as another non-SLE autoimmune disease. Therefore, elevated levels of IL-34 may be non-specific features of systemic inflammation and not a specific pathogenic factor of lupus. Further studies on IL-34 and other inflammation diseases are needed.

Both SLE and RA are systemic autoimmune diseases. Although functional studies are clearly required to confirm the role of IL-34 in the pathogenesis of SLE, the observed correlations between the elevated levels of IL-34 and SLEDAI, antibody production and anemia suggest that IL-34 plays a role in the modulation of immune inflammatory pathways in SLE.

Many cytokines play important roles in SLE^18^. TNF-α and IL-6 are major inflammatory mediators of pathology in both SLE and RA^18,19^. TNF-α also plays a role in anemia. Furthermore, IL-6^10,11^, IP10 ^12^ and MCP-1^13^ participate in the pathogenesis of SLE. IL-34 can induce the expression of IL-6, IP10 and MCP-1 in human whole blood^7^. The expression of IL-34 increases with inflammation in patients with inflammatory bowel disease and experimental colitis and is associated with an elevated TNFα and IL-6 expression^20^. Altogether, we hypothesize that IL-34 might play a role in the pathogenesis of SLE by promoting the production of other pro-inflammatory cytokines, including TNF-α, IL-6, IP10 and MCP-1.

In summary, the serum IL-34 level was significantly elevated in the SLE patients and correlated with the disease activity and homological changes. IL-34 could be a potential disease activity marker, and this study might have revealed new insight for the study of SLE disease activity.

## Methods

### Patients

This study was approved by the ethics committee of the 1^st^ Affiliated Hospital of China Medical University. The experiments were conducted in accordance with the regional Ethics Committee guidelines and regulations. Informed consent forms were obtained from all patients.

Serum was obtained from 110 patients with SLE and 31 patients with discoid lupus erythematosus (DLE), which is the most common chronic cutaneous lupus subtype. The diagnosis of SLE was based on the American College of Rheumatology criteria for SLE^21^ In addition, 55 healthy age- and gender-matched controls were recruited. All serum samples were stored at −70 °C until analysis. Since drugs can affect the circulating IL-34 levels, the serum was collected only from SLE patients who were not under treatment. Among the 110 SLE patients, eighty-nine had newly diagnosed SLE, and the other twenty-one were previously diagnosed but had not taken corticosteroids or immunosuppressive drugs for at least three months before enrollment in the study. All thirty-one DLE patients were newly diagnosed, and no drugs were used. Blood tests, including assays to determine C-reactive protein (CRP), complement (C) 3, C4, immunoglobulin (Ig) A, IgG, IgM, anti-double-stranded DNA antibody (Anti-dsDNA Ab) and hemoglobin (Hb) levels and white blood cell (WBC) and platelet (PLT) counts, were performed using standard methods. LN was identified if there was evidence of involvement according to the ACR renal criteria. The disease activity was scored using the SLE Disease Activity Index (SLEDAI)^22^.

### Measurement of IL-34 level in serum samples

The serum IL-34 level was measured using an enzyme-linked immunosorbent assay (ELISA) according to the manufacturer’s directions (R&D Systems, Minneapolis, MN). Each sample was measured in triplicate. Since serum antibodies can interfere with the assay for IL-34 and cause false positive results, a known quantity of recombinant IL-34 was added to the serum that contained the antibodies to determine whether the measurement was altered. No interference effect was detected (Supplementary Figure).

### Statistical analysis

All data are presented as the mean ± SD (parametric) or median (range) (non-parametric). The t-test and the non-parametric Mann-Whitney test were performed to compare the variables between groups. Pearson’s or Spearman’s correlation coefficient was used to test the correlations. A multivariable linear regression analysis was also performed to assess the independent predictive value of IL-34 in disease activity and hematological changes in the SLE patients. All analyses were performed using SPSS 17.0 and GraphPad 6 software.

## Electronic supplementary material

supplementary figure
