# Supplementary material for: Elevated Serum Interleukin-34 Level in Patients with Systemic Lupus Erythematosus Is Associated with Disease Activity
Source: Sci Rep. 2018 Feb 22;8:3462. doi: 10.1038/s41598-018-21859-z (PMC5823931; doi:10.1038/s41598-018-21859-z)
Supplement: Supplementary file 1 — supplementary figure [file 41598_2018_21859_MOESM1_ESM.pdf]

Supplementary figure

Elevated Serum Interleukin-34 Level in Patients with Systemic Lupus Erythematosus Is Associated with Disease Activity

Xie Huan Huan[1,2], Shen Hui[1], Zhang Li[1], Cui Mei Ying[1], Xia Li Ping[1], Lu Jing[1]

[1]Department of Rheumatology, 1st Affiliated Hospital of China Medical University, Shen Yang, China

[2]Department of Rheumatology, Dazhou Central Hospital, Da Zhou, China

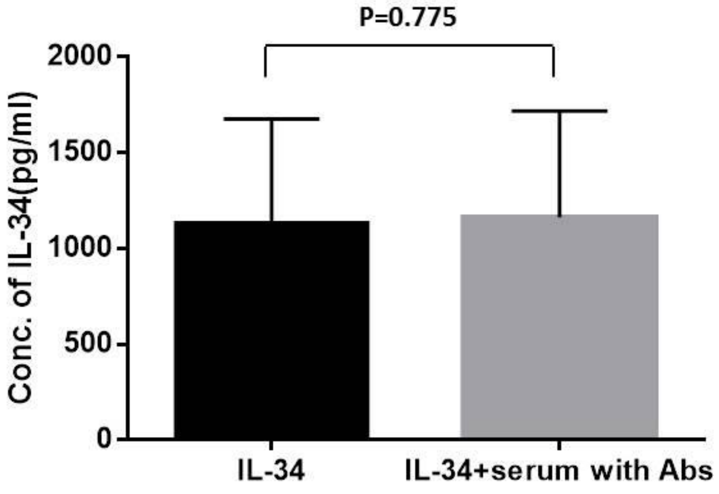

Heterophile antibody has no effect on IL-34 level.
